# New Cross-Linking Quinoline and Quinolone Derivatives for Sensitive Fluorescent Labeling

**DOI:** 10.1007/s10895-012-1039-z

**Published:** 2012-03-28

**Authors:** Shyamala Pillai, Maxim Kozlov, Salvatore A. E. Marras, Lev N. Krasnoperov, Arkady Mustaev

**Affiliations:** 1 Department of Chemistry and Environmental Sciences, New Jersey Institute of Technology, 151 Tiernan Hall, University Heights, Newark, NJ 07102 USA; 2 PHRI Center, New Jersey Medical School, Department of Microbiology and Molecular Genetics, University of Medicine and Dentistry of New Jersey, 225 Warren Street, Newark, NJ 07103 USA; 3 Enghelhardt Institute for Molecular Biology RAN Moscow, Vavilova 32, Moscow, 117984 Russia

**Keywords:** Quinoline, Quinolone, Fluorescence, Cross-linking probes

## Abstract

A variety of contemporary analytical platforms, utilized in technical and biological applications, take advantage of labeling the objects of interest with fluorescent tracers—compounds that can be easily and sensitively detected. Here we describe the synthesis of new fluorescent quinoline and quinolone compounds, whose light emission can be conveniently tuned by simple structural modifications. Some of these compounds can be used as sensitizers for lanthanide emission in design of highly sensitive luminescent probes. In addition, we also describe simple efficient derivatization reactions that allow introduction of amine- or click-reactive cross-linking groups into the fluorophores. The reactivity of synthesized compounds was confirmed in reactions with low molecular weight nucleophiles, or alkynes, as well as with click-reactive DNA-oligonucleotide containing synthetically introduced alkyne groups. These reactive derivatives can be used for covalent attachment of the fluorophores to various biomolecules of interest including nucleic acids, proteins, living cells and small cellular metabolites. Obtained compounds are characterized using NMR, steady-state fluorescence spectroscopy as well as UV absorption spectroscopy.

**Electronic supplementary material:**

The online version of this article (doi:10.1007/s10895-012-1039-z) contains supplementary material, which is available to authorized users.

## Introduction

Fluorescent labels are used in numerous applications that relay on sensitive detection of biological macromolecules (proteins, nucleic acids, polycarbohydrates, etc.), as well as for specific labeling of cells and tissues. In these applications, the fluorophore reporter groups are illuminated by visible or UV light, which leads to absorption of the light quantum and excitation of the molecule. The excited state is unstable and tends to relax either through dissipation of the absorbed energy by collision with other components in the medium or by emission of light. This light can be subsequently detected. Detection sensitivity is proportional to the number of the light quanta emitted by the fluorophore, which in turn is a linear function of the intensity of the excitation light. Therefore, for sensitive detection, high intensity light sources are employed. This creates the problem of discrimination of excitation and emission light, since even a small fraction of the excitation light that reaches the detector can cause significant background and decreases the detection sensitivity. This problem can be alleviated by using fluorophores with large spectral distances between excitation and emission light (Stokes shift). Quinolone [1] and quinoline fluorophores, discovered in the course of present study, possess the desired property. In this paper, we describe the synthesis and reaction mechanisms for new derivatives of these fluorophores that are suitable for attachment to biological macromolecules. We found that 7-aminoquinolones can be conveniently modified at either the 1-amido- or 2-oxogroup, yielding corresponding quinolone and quinoline derivatives with preserved fluorescent properties and large Stokes shift (ca. 50–110 nm). Subsequent modification of the resulting compounds at the 7-amino group allows tuning of the fluorescence from deep blue to green emission. Using analogous modifications, we synthesized amine-reactive isothiocyano-derivatives, as well as azido derivatives capable to click-react with acetylenic counterparts [for review see [2–4]. Reactivity of the compounds was verified in reactions with cysteine and alkyne-derivatized DNA oligos. The results suggest suitability of the new reactive probes for fluorescent labeling with detection limit in the nanomolar range.

## Results

### Investigation of the Reaction Mechanism for Quinolone and Quinoline Fluorophore Formation

**Scheme 1:**
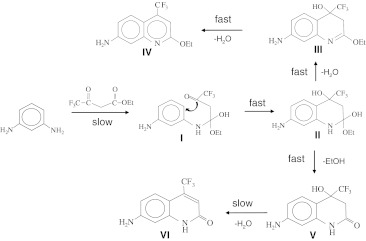
Reaction scheme between 1,3-phenylenediamine and 4,4,4-trifluoroacetoacetate. Structures I, II, and III are hypothetical

In our previous study [5], during the synthesis of carbostyril derivatives along with expected quinolone compound cs-124-CF_3_ [1], we detected a new fluorescent product which was not previously described. Since the compound was highly fluorescent and displayed large Stokes shift (120 nm), we set up to identify the compound and to study the reaction mechanism in more details in order to determine the influence of the reaction conditions on the product yield. The suggested mechanism for the reaction between ethyl 4,4,4-trifluoroacetoacetate with 1,3 phenylenediamine is shown in Scheme 1. Chromatographic analysis revealed that incubation of the starting compounds results in quick accumulation of an unknown fluorescent product (compound **IV** of Scheme 1, which we named Qin124-CF_3_) with R_f_ = 0.9, along with the expected compound cs124-CF_3_ (R_f_ = 0.44). Some non-fluorescent compounds, possibly reaction intermediates were also detected (compound **V** of Scheme 1, R_f_ = 0.62, and another product with R_f_ = 0.84). Continued incubation of these purified non-fluorescent products in the original reaction conditions showed that the compound **V** slowly converted into fluorescent cs124-CF_3_ (compound **VI** of Scheme 1, R_f_ = 0.44). At the same time, incubation of purified compound **V** in the reaction conditions did not lead to compound **IV**, suggesting that compound **IV** originates from an earlier reaction intermediate (possibly from compound **II**). This is consistent with the fact that fluorescent compound **IV** stops accumulating after intermediate **V** is completely formed in the course of the synthetic reaction (Fig. S1). Finally, the non-fluorescent reaction product with R_f_ = 0.84 was stable at the incubation conditions and therefore represented a side-product. For compound **II,** elimination of ethanol would lead to the observed precursor of cs124-CF_3_ (compound **V**), while dehydration would create compound **III**, which finally converts to fluorescent product **IV**. The identity of compounds **IV**, **V**, and **VI** was confirmed by NMR spectroscopy. In addition, we have shown that product **IV** was authentic to the compound obtained by O-ethylation of cs124-CF_3_ (see below) as judged by chromatographic mobility, UV absorption, fluorescence, and NMR spectroscopy. The formation of a similar derivative related to compound **IV** has been reported before, when 3-aminophenol was incubated with trifluoroacetoacetate to yield 7-hydroxyquinoline [6], reflecting a common reaction mechanism. The structure of the initial reaction intermediate is more uncertain, since the only stable adduct amenable for analysis is compound **V**. Ethyl 4,4,4-trifluoroacetoacetate has two electrophilic centers, which are carbons of carbonyl function and esterified carboxyl group. There are also two nucleophilic centers in phenylenediamine (amino groups and carbons of the ring in positions 4 and 6) that can be potentially attacked by acetoacetate derivative. Thus initial reaction can proceed through acylation of the amino group (compound **I**) followed by intramolecular attack of the phenyl ring (product **II**). As indicated by UV absorption spectroscopy and chromatographic mobility, the same fluorescent compounds were formed at both 50 °C and 110 °C. However, high temperature dramatically accelerates the formation of the quinolone derivative (from intermediate **V**), while the amount of the produced quinoline compound was not effected in accordance with proposed kinetic scheme (Scheme 1).

We found that compound **V**-to-compound **VI** conversion is sharply accelerated (ca. 10^6^ times) in the presence of NaOH. The same, but less pronounced effect (ca. 50 times) was observed upon the addition of a catalytic amount of trifluoroacetic acid to the starting reaction mixture. These findings allow dramatic reduction of both reaction temperature and the reaction time and nearly quantitative conversion of starting compounds to fluorophores **IV** and **VI**.

### Synthesis of Reactive Quinolone and Quinoline Derivatives (Chart 1)

**Scheme 2:**
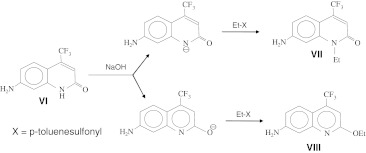
N- and O- alkylation of a quinolone fluorophore

**Scheme 3:**
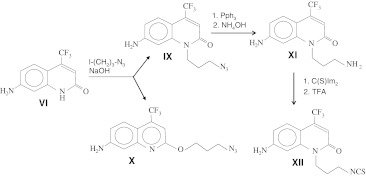
Synthetic route for quinoline and quinolone reactive derivatives

**Scheme 4:**
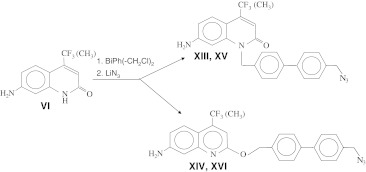
Synthesis scheme of cs124 reactive derivative with a biphenyl spacer

Our next goal was the synthesis of cross-linkable derivatives of cs124-CF_3_ and the newly discovered quinoline fluorescent derivative Qin124-CF_3_. To this end, we investigated the possibility of a chemical modification of these fluorophores. By analogy with previous observations [7, 8], we reasoned that the amide group of cs124-CF_3_ in ionized form can undergo alkylation, thus allowing introduction of cross-linking groups into the core moiety (Scheme 2). Indeed, incubation of cs124-CF_3_ with ethyl ester of p-toluenesulfonic acid in the presence of NaOH yielded two fluorescent products migrating with R_f_ = 0.44 and 0.9 on TLC, using an ethylacetate developing system. We proposed that these two products originate due to alkylation at the amido group nitrogen and oxygen that can assume a negative charge (providing high reactivity) as a result of lactim-lactam tautomery (see Scheme 2). These alkylation reactions would afford quinolone (**VII**) and quinoline (**VIII**) compounds, correspondingly. Indeed, NMR analysis confirmed the proposed structures. Next, we performed the same reaction, but with 1-iodo-3-azidopropane, alkylating compound bearing azido group (Scheme 3). In the other version we treated the fluorophore with bifunctional alkylating agent, 4,4′-bis(chloromethyl) biphenyl and then introduced the azido group by subsequent reaction with lithium azide. The final products (compounds **IX** and **X**) of Scheme 3 as well as compounds **XIII** and **XIV** of Scheme 4 can be used directly for coupling to the molecules of interest via the “click”-reaction with alkyne counterparts pre-attached to the molecules of interest. Alternatively, the azido group can be reduced to an amino group (compound **XI**) that can be converted to amine-reactive isothiocyano group (compound **XII**), or to thiol-reactive bromoacetamido group. The reduction of the azido-compound was performed with high yield by treatment with triphenylphosphine followed by incubation with ammonium hydroxide. Isothiocyano derivative was obtained by reaction of products **XI** with thiocarbonyldiimidazole, and subsequent treatment with trifluoroacetic acid. The reactivity of the resulting compounds was confirmed by reaction with cysteine using TLC analysis.

**Scheme 5:**
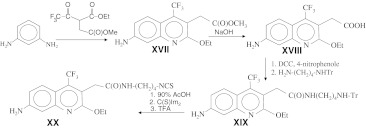
Synthetic strategy for a amine-reactive quinoline derivative

We also used similar approaches for the synthesis of biphenyl derivatives of 4-methylquinolones and 4-trifluoromethylquinolones (compounds **XIII** to **XVI)** (Scheme 4). The cross-linkable quinoline compounds could also be obtained using modified derivatives of ethyl 4,4,4-trifluoroacetoacetate by adapting the protocols published in our previous research for the synthesis of analogous quinolone compounds [5]. In this way, we first performed the alkylation of ethyl 4,4,4-trifluoroacetoacetate with methyl ester of bromoacetic acid at methylene carbon (Scheme 5). The resulting intermediate was incubated with 1,3-phenylenediamine at moderate temperature that favors formation of quinoline compound (product **XVII** of Scheme 5) that was converted to cadroxylate (compound **XVIII**) by saponification. This compound was treated with carbodiimide and 4-nitrophenole resulting in an activated ester that was introduced in reaction with mono-tritylated 1,4-diaminobutane yielding compound **XIX**. Deprotection followed by treatment of the resulting amino-derivative with N,N-thiocarbonyldiimidazole and trifluoroacetic acid afforded product compound **XX**, an isothiocyanate derivative.

### UV Absorption and Fluorescent Spectra of the Synthesized Compounds

**Fig. 1:**
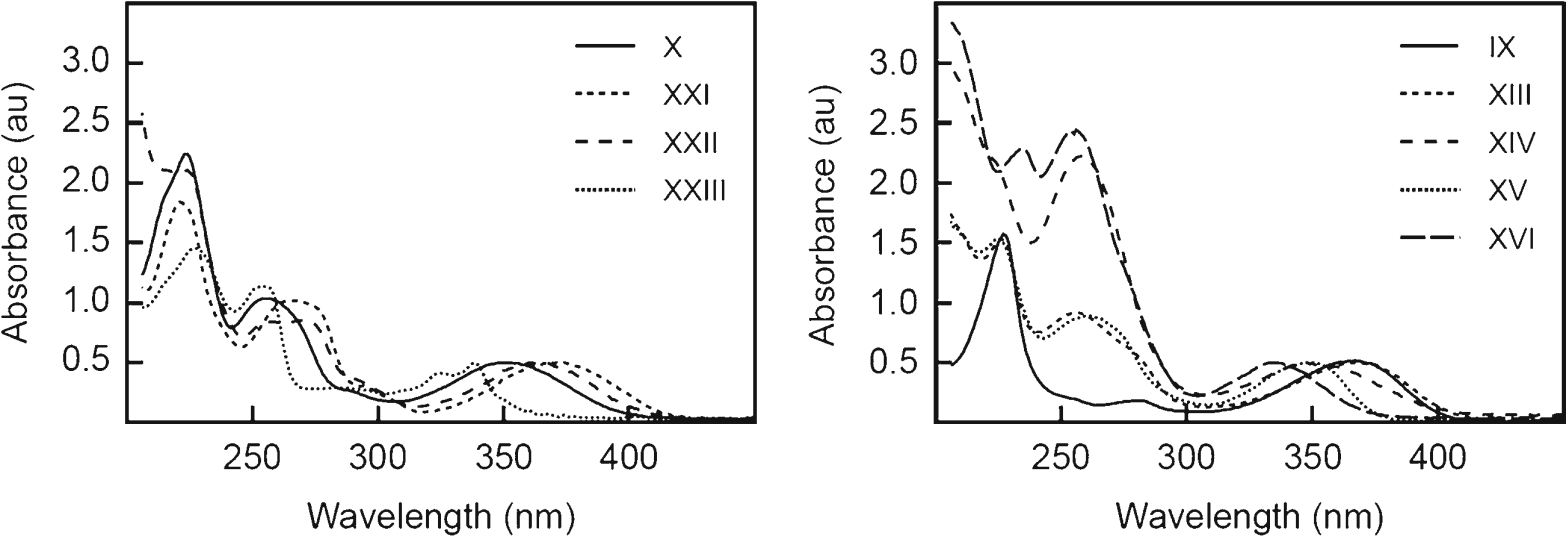
UV absorption spectra for reactive fluorophore compounds

As seen from Figure 1 and Table 1, the synthesized compounds have absorption maxima in the register 200–300 nm, as well as longer wavelength absorption (300–400 nm), which is optimal for fluorescence excitation. The molar extinction for the compounds in this far UV region vary from 6000 to ca. 19 000 M^−1^ sm^−1^. Generally, quinolone compounds in the range 300–400 nm have extinction coefficients two to three fold greater than corresponding quinoline compounds (compare compounds **IX** and **X**; **XIII** and **XIV**). Quantum yields for compounds **XIII** and **XIV** are comparable, while quantum yield for **X** is half of that for **IX**. Among corresponding quinolone and quinoline derivatives of 4- methyl substituted compounds, quantum yields differ insignificantly as well (compare comp. **XV** and **XVI**).

**Table 1:** Fluorescent and UV-absorption properties of synthesized fluorophores and reference compound, 4-methylumbelliferone

Compound	Absorption M-1 × cm-1 (εo)	Excitation (nm)	Emission (nm)	Stokes shift (nm)	Quantum yield* (Φ)	Brightness εo x Φ
4-Me-umb.	16 500	365	446	80	0.7	11 550
IX	18 900	370	450	104	0.26	4 914
X	10 000	359	463	51	0.47	4 700
XV	18 000	353	404	70	0.51	9 180
XVI	9 000	343	413	78	0.39	3 150
XIII	18 000	372	450	101	0.54	9 720
XIV	6 000	360	461	98	0.42	2 520
XXI	10 000	367	465	107	0.28	2 800
XXII	10 000	374	481	63	0.27	2 700
XXIII	10 000	339	402	81	0.14	1 400

**Fig. 2:**
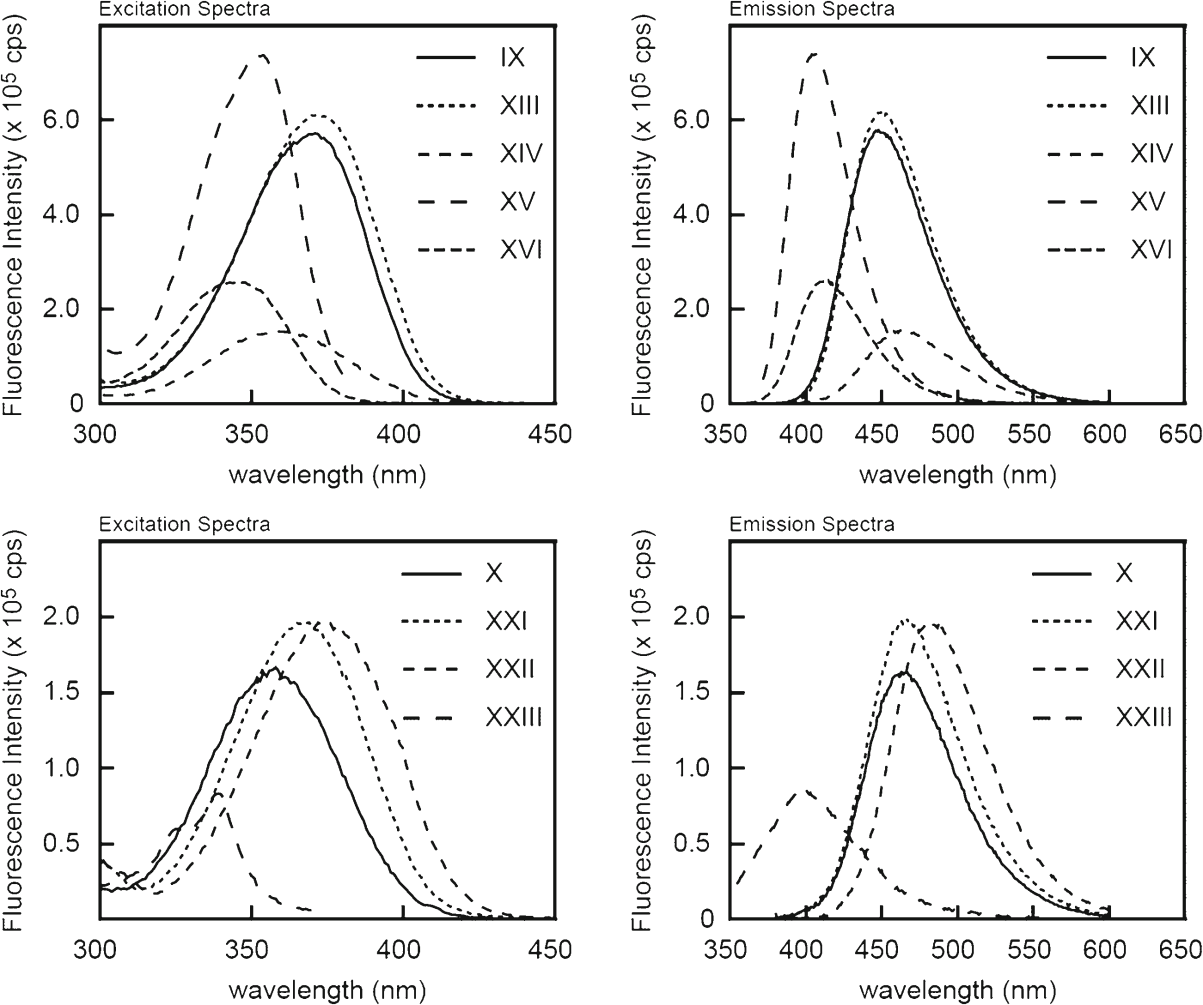
Fluorescent spectra for reactive fluorophore compounds. **a**, **b**—excitation spectra; **c**, **d**—emission spectra

**Fig. 3:**
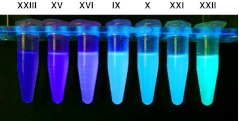
Fluorescence of the reactive fluorophore compounds

In order to generate fluorophores with various colors, we examined how modification of 7-amino group of quinoline compounds effect their emission spectrum. Thus we obtained mono- and dimethylamino- derivatives (compounds **XXI** and **XXII** correspondingly) and related acetamido- derivative (compound **XXIII**). As seen from Table 1 and Figure 2 these modifications significantly changed fluorescent properties of the original fluorophore (compound **X**). Thus methylation of the aminogroup caused red shift, while acylation resulted in strong blue shift of the emission spectrum. While methylation did not affect quantum yield of the fluorophore, acetylation caused a two fold reduction in quantum yield. Comparing to trifluoromethyl fluorophores (compounds **IX** and **X**) corresponding 4-methyl derivatives (compounds **XV** and **XVI**) displayed a blue shift of the fluorescence emission (Table 1 and Fig. 2). Generally, Stokes shifts were larger for 4-trifluoromethyl compounds comparing to the corresponding 4-methyl fluorophores. The emission spectra of the synthesized reactive fluorescent compounds cover all colors from deep blue to green (Figs. 2 and 3), which makes them very useful as labels in biochemical and technical applications.

**Chart 1:**
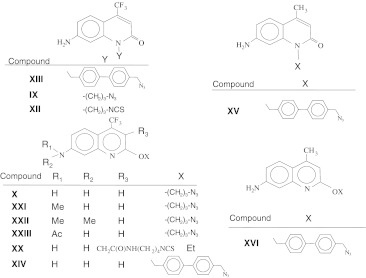
Structures of the synthesized reactive fluorophore derivatives

### Chemical Reactivity of Synthesized Compounds

In the present study, we obtained amine and thiol-reactive isothiocyano (ITC), as well as azido-derivatives of fluorophores, which are suitable for click-reaction with acetylenic counterparts, catalyzed by copper complexes. The reactivity of ITC compounds was examined with cysteine at weakly alkaline conditions favoring ionization of thiol group. The reaction proceeded quickly and quantitatively at room temperature, yielding dithiocarbamate derivatives as was evidenced by strongly reduced mobility of the reaction products on TLC. The same effect was observed in reaction of ITC compounds with ethylenediamine at 50 °C.

Click reactivity of azido-fluorophores was confirmed by incubation with alkyne-derivatized oligonucleotides in the presence of copper complex and ascorbate. HPLC analysis of the reaction mixture revealed nearly quantitative coupling of the fluorophores to the oligos (Fig. S2). These results suggest suitability of the synthesized compounds for fluorescent DNA labeling. It should be mentioned that attachment of fluorophores to DNA was accompanied by considerable quenching (Table 2), which was most likely due to stacking interaction of the fluorophores with DNA bases.

**Table 2:** Fluoroscence of free fluorophores and the same fluorophores attached to DNA

Probe	Fluorescence of free probe	Fluorescence of DNA-bound probe	Quenching factor
XIII	5.9 × 105	2.5 × 105	2.4
XV	7.5 × 105	1.1 × 105	6.8
IX	5.8 × 105	1.6 × 105	3.6

## Conclusion

In the present research, we explored new synthetic strategies to obtain fluorescent derivatives of quinolone fluorophores. In the course of optimization of the reaction conditions for the synthesis of 4-trifluoromethyl quinolone compound, we observed a previously unknown product with useful fluorescent properties. This prompted us to investigate the reaction mechanism in more details. NMR spectrum analysis, along with kinetic data of the product accumulation suggested that this new compound represents 7-amino-4-trifluoromethyl-2-ethoxyquinoline. The identity of the product was confirmed by its independent synthesis route through ethylation of 7-amino-4-trifluoromethylquinolone. Further characterization of this compound (Table 1) revealed its valuable fluorescent properties, comprising a large Stokes shift (104 nm) and a high quantum yield (ca. 0.3).

Next, we explored the possibility to modify quinolone compounds with the aim to introduce cross-linkable groups for fluorescent labeling. We observed that quinolones can be easily modified by alkylation either at the amide nitrogen, or oxygen to yield N-1 derivatives of quinolone, or O-2 derivatives of quinoline, correspondingly. The reaction proceeds with a high yield under alkaline conditions that promote ionization of the amido group. Using this reaction, we further obtained cross-linkable derivatives containing isothiocyano-, or azido groups. In addition, we explored alternative ways to introduce cross-linking group into quinoline moiety using a previously described reaction of 1,3-phenylenediamine with trifluoromethylmethylethylsuccinate that proved useful for corresponding quinolone derivatives. The desired compound was obtained with reasonable yield by optimization of the reaction temperature. Structurally related 4-methyl quinolone and quinoline fluorescent derivatives were obtained using analogous derivatization reactions. These derivatives exhibit considerable blue emission shift compared to 4-trifluoromethyl counterparts. The obtained fluorescent compounds can be further modified at the 7-amino group, allowing tuning of fluorescence emission, so that altogether the synthesized reactive compounds span emission register from deep blue to green. These fluorescent compounds can be efficiently cross-linked to DNA and proteins, which makes them valuable probes for biochemical applications with detection limit in the nanomolar range.

## Experimental Section

### Materials and Methods

The following reagents were purchased from Aldrich: 1,3-diiodopropane, triphenylphosphine, triethylamine, 1,3-phenylenediamine, ethyl 4,4,4-trifluoroacetoacetate, p-toluenesulfonylchloride, *N*-trityl-1,6-diaminohexane, 4,4′-bischloromethylbiphenyl, methylbromacetate, anhydrous dimethylformamide and dimethylsulfoxide, 1-butanol, ethylacetate, chloroform, acetonitrile, ethanol, sodium, potassium, and ammonium hydroxide, lithium azide, Na_2_SO_4_, Na_2_CO_3_, acetic acid, citric acid, thiocarbonyldiimadazole, TBTA-copper complex, TbCl_3_, EuCl_3_, SmCl_3_ and DyCl_3_, silica gel TLC plates on aluminum foil (200 μm layer thick with a fluorescent indicator). All chemicals were the purest grade available. Excitation and emission fluorescence spectra in a steady-state mode were recorded using a Quanta-Master 1 (Photon Technology International) digital spectrofluorometer at ambient temperature. UV-absorption spectra were recorded on a UV-visible spectrophotometer (Cary 300 Bio, Varian). 2′-O-methyl-RNA oligos containing the following sequences were used: 5′ hexynyl—CUUC GUC CAC AAA CAC AAC UCC U GAAG—BHQ2 3′; 5′hexynyl—CUAG ACC ACA CAA CCU AC CUAG—BHQ2 3′; 5′ hexynyl—GCC UCG UCG CCG CAG CUA ACU AUC CGU GUG CGUC—NH_2_ 3′.

### Synthesis

1. 7-amino-4-trifluromethyl-quinolone (cs124-CF_3_, compound VI, Scheme 1), improved method and 7-amino-4-trifluromethyl-2-ethoxy quinoline (Qin124-CF_3_ , compound IV)
  1.1 Improved protocol.
    Twenty mmol (2.16 gm) of 1,3-phenylenediamine dissolved in 10 ml DMSO were mixed with 20 mmol (3.68 gm) of ethyl 4,4,4-trifluoroacetoacetate. The mixture was incubated 1.5 h at 80 °C, supplemented with 2 ml of 10 M aqueous KOH and kept for another 20 min at room temperature. Two fluorescent products with R_f_ = 0.92 (green blue) and 0.44 (blue) were detected by TLC in ethylacetate. The mixture was poured into 10 ml of 0.1 M citric acid and left for 2 h on ice. The precipitate (cs124-CF_3_) and Qin124-CF_3_ was collected by filtration, washed with water, dried and suspended in chloroform. After filtration the precipitate (pure cs124-CF_3_) was dried under reduced pressure, while chloroform filtrate containing mostly fluorescent product with high mobility (Qin124-CF_3_) on TLC was evaporated *in vacuo* and the product purified by silicagel column chromatography using hexane-acetone (3:1) as eluent. The fractions containing pure product were collected and evaporated to dryness. Yields: cs124CF_3_ ~ 44 % and for Qin124-CF_3_ ~ 26 %. UV absorption spectrum for cs-124-CF_3_: λ_max1_ = 230 nm and λ_max2_ = 270 nm λ_max3_ = 360; For Qin-124: λ_max1_ = 226 nm and λ_max2_ = 315 nm, λ_max3_ = 345 nm. 1H NMR for Qin124-CF_3_ : 7.60 (dd,1H, 5H, J_1_ = 9.0, J_2_ = 2.1), 6.94 (dd, 1H, 6H, J_1_ = 9.0, J_2_ = 2.4), 6.88 (s, 1H. 8H), 6.85 (d, 1H, 3H, J = 2.1), 6.0 (s, 2H, 7-amino), 4.42 (q, 2H, -OCH_2_, J = 7.2), 1.35 (t, 3H, methyl, J = 7.2)
  1.2 Effect of incubation conditions on product distribution in the reaction of 1.1 In order to confirm the structure of reaction products and the reaction intermediates, 108 mg (1 mmol) of 1,3-phenylenediamine was dissolved in 0.5 ml DMSO and supplemented with 150 μl of ethyl 4,4,4-trifluoroacetoacetate. The reaction mixture was divided into equal portions ca. 370 μl each. One portion was incubated at 50 °C for 3 h, while the other half was incubated at 110 °C for 1 h. One hundred microliter aliquots of the reaction mixtures diluted with 250 μl of n-butanol were applied on TLC plates and dried in hot air flow. After separation in ethyl acetate as developing solvent, the fluorescent products with R_f_ = 0.44 (cs124-CF_3_) and R_f_ = 0.92 (Qin124-CF_3_) along with non-fluorescent products with R_f_ = 0.62 and 0.84 were eluted by methanol and evaporated to dryness. UV absorption spectrum for cs124-CF_3_: λ_max1_ = 360 nm. For Qin124-CF_3_: λ_max_ = 347 nm. For bottom Intermediate: λ_max_ = 230 nm. For top intermediate: λ_max_ = 310 nm. ^1^H NMR spectra for the products with R_f_ = 0.44 and 0.92 were identical to cs124-CF_3_ and Qin124-CF_3_ correspondingly (see previous section). ^1^H NMR for the product with R_f_ = 0.62 (comp. **V**): 10.15 (s, 1H, -NH), 7.15 (d, 1H, 6H, J = 8.4) , 6.57 (s,1 H, 8H), 6.24 (dd, 1H, 5H, J_1_ = 8.3 , J_2_ = 2.1), 6.10 (d, 1H, hydroxy H, J = 2.1), 5.4 (s,2H, 7-amino), 2.76 (q, 2H, 3H, J_1_ = 32.4 , J_2_ = 16.5)
  1.3 Incubation of non-fluorescent reaction products of 1.2 at various conditions.
    The solutions of purified reaction products with R_f_ = 0.62 and 0.84 (see previous section) in DMSO (ca. 1 mg/ml, 100 μl,) were kept either at 110 °C or supplemented with 2 μl of 10 M KOH and left at room temperature. At time intervals the mixtures were analyzed by TLC in ethylacetate as developing solvent.
2. Alkylation of cs-124CF_3_ by ethyl p-toluenesulfonate.
  Solution of 300 μmol of cs124-CF_3_ in 500 μL DMSO was supplemented with 16.8 mg of anhydrous powdered KOH and 400 μmol of ethyl p-toluenesulfonate. The reaction mixture was vortexed for 30 min and left for 4 h until completion of the reaction as confirmed by TLC in hexane-acetone (3:1) system. Two reaction products were observed. The reaction mixture was poured dropwise to 12 ml water. After centrifugation at 6000 rpm for 10 min, the precipitate was collected and dissolved in 1 ml of ethanol and subjected to TLC in hexane-acetone (3:1) solvent system. Product with R_f_ = 0.38 was eluted by methanol and evaporated *in vacuo.* UV absorption spectrum was identical to comp. **IV** (see above). Yield = 5.7 mg. ^1^H NMR: 7.60 (dd,1H, 5H, J_1_ = 9.0, J_2_ = 2.1), 6.93 (dd, 1H, 6H, J_1_ = 9.0, J_2_ = 2.4), 6.88 (s, 1H. 8H), 6.85 (d, 1H, 3H, J = 2.1), 6.0 (s, 2H, 7-amino), 4.42 (q, 2H, -OCH_2_, J = 7.2), 1.35 (t, 3H, methyl, J = 7.2)
3. 1-(3-azidopropyl)-cs124-CF_3_ (compound IX), 7-amino-4-trifluoromethyl-2-(3-azidooxypropyl)quinoline compound X) and 1-(3-isothiocyanopropyl)-cs124-CF_3_ (compound XII).
  3.1 1-iodo-3-azido propane.
    Nine grams of 1,3-diiodopropane were mixed with 15 ml of DMF and supplemented with 1.5 g of lithium azide. After 1 h incubation at 37 °C, TLC analysis using hexane as developing solvent revealed one reaction product with R_f_ = 0.2 along with starting 1,3-diiodopropane (R_f_ = 0.4). The mixture was poured into 150 ml of water and extracted with an equal volume of ether. The organic layer was dried over anhydrous sodium sulfate and evaporated under reduced pressure. The residue was dissolved in hexane and subjected to silicagel column chromatography using the same solvent as eluent. The fractions corresponding to product were collected and evaporated to dryness. Yield = 2.4 g, (30 %).
  3.2 1-(3-azidopropyl)-cs124-CF3 (compound IX) and 7-amino-4-trifluoromethyl-2-(3-azidooxypropyl) quinoline (compound X).
    Four hundred fifty six milligrams of cs124-CF_3_ dissolved in 4 ml of DMSO were supplemented with 112 mg of anhydrous KOH and 422 mg of IC_3_H_6_N_3_ and left overnight at room temperature. TLC analysis in hexane-acetone (3:1) mixture revealed the presence of two fluorescent reaction products, R_f_ = 0.15 (blue fluorescence, azido cs124-CF_3_) and R_f_ = 0.3 (green-blue fluorescence, azido Qin124-CF_3_). The reaction products were precipitated by addition of 45 ml of water, dried under *vacuo,* dissolved in acetone and purified by silica gel column chromatography using hexane-acetone (3:1) as eluent. The fractions corresponding to the products migrating to R_f_ = 0.15 and 0.3 were collected and evaporated to dryness. 1-(3-azidopropyl)-cs124-CF_3_ (compound **IX**): Yield = 292 mg, λ_max1_ = 281 nm, UV absorption spectrum: λ_max2_ = 369 nm (ε = 17150 M^−1^ cm^−1^). ^1^H NMR chemical shifts (d) in DMSO are: 7.43 (dd, 1H, 5H, J = 8.7), 6.62 (m, 2H, 6H and 8H combined), 6.55 (s, 1H, 3H), 6.22 (s, 2H, 7-amino), 4.18 (t, 2H, α-CH_2_, J = 7.2), 3.48 (t, 2H, γ-CH_2_, J = 6.75), 1.86 (quintet, 2H, β-CH_2_, J = 6.9). Compound **X**: Yield = 84 mg, UV absorption spectrum:λ_max1_ = 255 nm, λ_max2_ = 350 nm (ε = 12300 M^−1^ cm^−1^ ^1^H NMR chemical shifts (d) in DMSO are: 7.67 (dd, 1H, 5H), J = 1.8), 6.92–6.96 (dd, 1H, 6H, J = 2.4), 6.9 (s, 1H, 8H), 6.86 (d, 1H, 3H, J = 2.1), 4.4 (t, 2H, γ-CH_2_, J = 6.3), 3.5(t, 2H, α-CH_2_, J = 6.6), 2.0 (quintet, 2H, β-CH_2,_ J = 6.4).
  3.3 1-(3-aminopropyl)-cs124-CF3 (compound XI).
    Two hundred milligrams of azido cs124-CF_3_ were treated with 217 mg of triphenylphosphine in 1 ml DMF at 50 °C for 2.5 h followed by addition of 0.2 ml of ammonium hydroxide and the incubation continued at the same temperature. After 1 h the reaction mixture was acidified with 1 M citric acid to pH ~ 4.0–4.5 and extracted with ether (2 × 10 ml). The pH of the aqueous phase was adjusted to pH 10 by NaOH and the solution was extracted with ester (3 × 20 ml). The ether extracts were collected, dried over anhydrous sodium sulfate and evaporated *in vacuo*. The residue was further dissolved in acetone and evaporated *in vacuo* to afford crystalline product. Yield = 183 mg (85 %). ^1^H NMR chemical shifts (d) in DMSO are: 7.44(dd, 1H, 5H, J = 8.7), 6.71(s, 1H, 8H), 6.65(dd, 1H, 6H, J = 8.8), 6.56(s, 1H, 3H), 6.24(s, 2H,7-amino), 4.18(t, 2H, α-CH_2_, J = 7.2), 3.24(t, 2H, terminal amine, J = 6.4), 2.6(t, 2H, γ-CH_2_, J = 6.6), 1.73(quintet, 2H, β-CH_2_, J = 6.9).
  3.4 1-(3-isothiocyanopropyl)-cs124CF_3_ (compound XII).
    The solution of 28.4 mg of compound **XI** (see precious paragraph) in 0.3 ml of DMSO was mixed with 21.4 mg of 1,1′-thiocarbonyldiimidazole in 0.2 ml of chloroform and incubated at ambient temperature. After 10 min, 3 μl TFA were added and the incubation continued for 1 h at 50 °C. TLC in hexane-ethylacetate (2:1) mixture as developing solvent revealed near complete conversion to isothiocyanate compound. The product was precipitated by 8 ml of water, the residue dissolved in 1.5 ml of methanol, re-precipitated by another 8 ml water, and after centrifugation at 7000 rpm for 5 min dissolved in 2.0 ml of methanol and dried *in vacuo*. Yield = 18 mg (55 %) yellow powder. ^1^H NMR chemical shifts (d) in DMSO are: 7.45(d, 1H, 5H, J = 8.7), 6.68(s, 1H, 6H), 6.65(s, 1H, 8H,), 6.57(s, 1H, 3H), 6.19(s, 2H,7-amino), 4.25(t, 2H, γ-CH_2_, J = 6.9), 3.78(t, 2H, α-CH_2_, J = 6.4), 2.04(quintet, 2H, β-CH_2_, J = 6.6).
  3.5 7-amino-4-trifluoromethyl-2-[1-methyleno(4-p-methylazido)biphenyl]quinolone (compound XIII) and 7-amino-4-trifluoromethyl-2-O-[methyleno(4-methylazido)biphenyl]quinoline (compound XIV.)
    Solution of 53 mg of cs124-CF_3_ in 0.4 ml of DMF was consequently mixed with 36 μl of aqueous 10 M NaOH and a solution of 150 mg of 4,4′-bischloromethylbiphenyl in 1 ml DMF. After 20 min incubation at room temperature, 50 mg of lithium azide was added and incubation continued for another 20 min at 60 °C. TLC analysis in hexane-acetone 2:1 system revealed two main products with relative mobility of 0.35 (for **XIII**) and 0.45 (for **XIV**). The reaction mixture was poured in 10 ml of water, the residue precipitated by centrifugation, washed with water, dissolved in 10 ml of acetonitrile and evaporated to dryness under reduced pressure. The products were purified by silicagel column chromatography in the same system. Yield for compound **XIII** was 60 mg; for compound **XIV**—10 mg. UV absorption spectrum, comp. **XIII**: λ_max1_ = 226 nm, λ_max2_ = 257 nm, λ_max3_ = 368 nm. λ_min1_ = 218 nm, λ_min2_ = 241 nm, λ_min3_ = 308 nm. For comp. **XIV**: λ_max1_ = 258 nm, λ_max2_ = 347 nm, λ_min1_ = 238 nm, λ_min2_ = 307 nm. ^1^H NMR chemical shifts (d) in DMSO for comp. **XIII** are: 7.66 (m, 4H, o,o’biphenyl H), 7.48 (dd overlapped, 1H, 5H, J = 2.1), 7.45 (d, 2H, biphenyl m-H, J = 8.1), 7.3 (d, 2H, biphenyl-m’- H, J—8.4), 6.7 (s, 1H, 8H), 6.62 (dd, 1H, 6H, J_1_ = 9.0, J_2_ = 1.8), 6.55 (d, 1H, 3H, J = 1.8), 6.25 (s, 2H, 7 amino), 5.45 (s, 2H, -CH_2_-N_3_), 4.48 (s, 2H, N-CH_2_). For comp. **XIV**: 7.71 (dd, 4H, o,o’biphenyl H, J_1_ = 8.25, J_2_ = 2.4), 7.65 (dd overlapped, 1H, 5H, J_1_ = 11.85, J_2_ = 2.4), 7.61 (d, 2H, biphenyl m-H, J = 8.1), 7.47 (d, 2H, biphenyl-m’- H, J—8.1), 6.97 (d, 1H, 6H, J = 2.1), 6.94 (d, 1H, 8H, J = 2.1), 6.9 (d, 1H, 3H, J = 2.1), 6.04 (s, 2H, 7 amino), 5.5 (s, 2H, -CH_2_-N_3_), 4.5 (s, 2H, O-CH_2_).
  3.6 7-amino-4-methyl-2-[1-methyleno(4-p-methylazido)biphenyl]quinolone (compound XV) and 7-amino-4-methyl-2-O-[methyleno(4-p-methylazido)biphenyl]quinoline (compound XVI).
    These compounds were synthesized as corresponding trifluoromethyl-derivatives (see above). R_f_ for **XV** is 0.22 (in hexane-acetone 2:1 developing system). UV absorption spectrum for comp. **XV:** λ_max1_ = 225 nm, λ_max2_ = 260 nm, λ_max3_ = 350 nm, λ_min1_ = 217 nm, λ_min2_ = 243 nm, λ_min3_ = 308 nm; for comp. **XVI**: λ_max1_ = 235 nm, λ_max2_ = 256 nm, λ_max3_ = 335 nm, λ_min1_ = 225 nm, λ_min2_ = 242 nm, λ_min3_ = 305 nm. ^1^H NMR chemical shifts (d) in DMSO for comp. **XV** are: 7.65 (dd, 4H, o,o’biphenyl H, J_1_ = 11.1, J_2_ = 8.4 ), 7.45 (dd overlapped, 1H, 5H), 7.45 (dd,2H, biphenyl m-H, J_1_ = 8.25, J_2_ = 5.1), 7.25 (d, 2H, biphenyl-m’- H, J—8.1), 6.49 (d, 1H, 6H), 6.44 (dd, 1H, 3H, J = 1.8), 6.21 (s, 1H, 8H), 5.8 (s, 2H, 7-amino), 5.38(s, 2H, N-CH_2_), 4.4 (s, 2H, -CH_2_-N_3_), 2.36 (d, 3H, 4-methyl, J = 0.9).
  3.7 7-methylamino-4-trifluoromethyl-2[1-(3-azidooxypropyl)]quinoline (compound XXI) and 7-dimethylamino-4-trifluoromethyl-2[1-(3-azidooxypropyl)])quinoline (compound XXII).
    Ten milligrams of 7-amino-4-trifluoromethyl-2-(3-azidooxypropyl) quinoline were dissolved in 0.1 ml of DMF and supplemented with 30 μl of iodomethane. After 1 h, 2 μl of N-,N-diisopropylethylamine was added and incubation continued for another 4 h. The alkylation products were purified by preparative TLC using hexane-acetone (3:1) mixture as developing solvent. Yield for compound **XXI** was 1 mg: UV absorption spectrum: λ_max1_ = 257 nm, λ_max2_ = 271 nm, λ_max3_ = 360 nm. λ_min1_ = 246 nm, λ_min2_ = 262 nm, λ_min3_ = 315 nm. Yield for compound **XXII** was 2.5 mg. UV absorption spectrum, λ_max1_ = 221 nm, λ_max2_ = 266 nm, λ_max3_ = 378 nm. λ_min1_ = 208 nm, λ_min2_ = 246 nm, λ_min3_ = 318 nm. ^1^H NMR chemical shifts (d) in DMSO for comp. **XXI** are: 7.60 (dd,1H, 5H, J_1_ = 9.15, J_2_ = 2.1), 6.97 (dd, 1H, 6H, J_1_ = 9.15, J_2_ = 2.1), 6.90 (s, 1H. 8H), 6.85 (d, 1H, 3H, J = 2.1), 6.69 (d, 1H, 3H,J = 2.4), 6.64 (q, 1H, 7-amino, J = 5.1), 4.46 (t, 2H, γCH_2_, J = 6.3), 3.54 (t, 2H, αCH_2_, J = 6.6), 2.79 (d,3H, monomethy-NCH_3_, J = 4.8, 2.05 (quintet, 2H, βCH_2_, J = 6.6). For comp. **XXII**: 7.74 (dq,1H, 5H, J_1_ = 5.1, J_2_ = 2.1), 7.2 (dd, 1H, 6H, J_1_ = 9.15, J_2_ = 2.7), 6.99 (s, 1H. 8H), 6.9 (d, 1H, 3H, J = 2.7), 4.48 (t, 2H, γCH_2_, J = 6.3), 3.54 (t, 2H, αCH_2_, J = 6.6), 3.07 (s, 6H, monomethyl and dimethyl group), 2.06 (quintet, 2H, βCH_2_, J = 6.3).
  3.8 7-acetamido-4-trifluoromethyl-2-(3-azidooxypropyl) quinoline (compound XXIII).
    Five milligrams of compound **X** were dissolved in 50 μl of DMF and 20 μl of acetic anhydride was added. The reaction was allowed to proceed at room temperature for 1 h. The product was purified by preparative TLC using hexane-acetone (3:1) mixture. Yield 4.2 mg. UV absorption spectrum: λ_max1_ = 226 nm, λ_max2_ = 254 nm, λ_max3_ = 325 nm, λ_max4_ = 339 nm; λ_min1_ = 241 nm, λ_min2_ = 302 nm, λ_min3_ = 330 nm. ^1^H NMR chemical shifts (d) in DMSO for comp. **XXIII** are: 10.43 (s, 1H, -NH), 8.42 (d, 1H, 8H, J = 1.8), 7.9 (d,1H, 5H, J_1_ = 9.1, J_2_ = 2.1), 7.61 (dd, 1H, 6H, J_1_ = 9.0, J_2_ = 2.1), 7.23 (s, 1H. 3H), 4.52 (t, 2H, γCH_2_, J = 9.6), 3.56 (t, 2H, αCH_2_, J = 6.6), 2.13 (s, 3H, acetyl methyl group), 2.06 (quintet, 2H, βCH_2_, J = 6.3).
  3.9 7-amino-4-ethoxy-3-carboxamido(6-isothiocyanobutyl)-2-trifluoromethylquinoline (XX).
    3.9.1 1, 7-amino-4-ethoxy-3-carbomethoxymethyl-2-trifluoromethylquinoline (XVII)
      A mixture of 2.2 ml trifluoroacetoacetate and 1.5 ml methylbromoacetate in 7 ml DMF was placed in a round-bottom flask, cooled on ice and 1.0 g of powdered KOH was added in a few portions under intensive agitation so that the temperature of the reaction mixture is kept in the range 40–50 °C. After 1 h reaction the incubation continued at 60 °C for another 1 hr. The reaction mixture was then diluted with 20 ml water and extracted with chloroform. The organic layer was collected, dried over anhydrous sodium sulfate and evaporated *in vacuo* at 70 °C for 30 min to remove un-reacted components. The residue (1.9 g) was dissolved in 3.5 ml of DMSO and 0.76 g of 1,3-phenylenediamine was added, followed by incubation at 50 °C overnight. A major fluorescent product (R_f_ = 0.9 in ethylacetete as developing solvent) with intense green-blue fluorescence was observed. The mixture was diluted to 30 ml by 0.05 M aqueous NaOH and extracted with ether (2 × 40 ml). The organic layer was dried over anhydrous sodium sulfate and evaporated *in vacuo*. The residue was then subjected to silica gel chromatography on 40 ml column using hexane-acetone (4:1) mixture as eluent. The fraction corresponding to the products migrating with R_f_ = 0.9 in ethylacetete was collected and evaporated to dryness. Yield 400 mg (12 %). UV absorption spectrum: λ_max_ = 350 nm (ε = 10000 M^−1^ cm^−1^), λ_min_ = 270 nm (ε = 6300 M^−1^ cm^−1^). ^1^H NMR chemical shifts (d) in DMSO are: 1,33(t, 3H, 4-OCH_2_C**H**_3_, J = 7.2), 3.65 (s, 3H,-OC**H**_3_), 3.94 (q, 2H, 3-methylene, J = 2.4), 4.38 (q, 2H, 4-OC**H**_2_CH_3_, J = 7.2), 5.94 (2H, broad, 7 amine), 6.83 (d, 1H, 8H, J = 2.4), 6.92 (dd, 1H, 6H), 7.67 (m, 1H, 5H).
    3.9.2 7-amino-4-ethoxy-3-carboxymethyl-2-trifluoromethylquinoline (XVIII).
      One milliliter of 1 M aqueous NaOH was added to 100 mg of product **XVII** dissolved in 2 ml dioxane. After 2 h of incubation at 50 °C, the organic solvent was evaporated at reduced pressure. The aqueous solution was acidified to pH 3–3.5 by addition of citric acid and extracted with chloroform. The chloroform layer was collected, dried over anhydrous sodium sulfate and evaporated *in vacuo*. Yield = 60 mg. ^1^H NMR chemical shifts (d) in DMSO are: 1,34 (t, 3H, 4-OCH_2_C**H**_3_, J = 7.2), 3.84, (q, 2H, 3-methylene, J = 2.4), 4.41 (q, 2H, 4-OC**H**_2_CH_3_, J = 7.2), 5.9 (2H, broad, 7 amine), 6.83 (d, 1H, 8H, J = 2.4), 6.92 (dd, 1H, 6H, J_1_ = 10, J_2_ = 2.4), 7.67 (m, 1H, 5H), 12,52 (1H, broad, carboxyl).
    3.9.3 7-amino-4-ethoxy-3-methylcarboxamido(4-tritylaminobutylmethyl)-2-trifluoromethylquinoline (XIX).
      Compound **XVIII** (60 mg, 180 μmol) was dissolved in 2 ml of THF and supplemented with 28 mg (200 μmol) of 4-nitrophenol and 100 mg (380 μmol) of dicyclohehylcarbodiimide (DCC). Following 30 min incubation, 300 μmol of N-trityl-1,4-diaminobuthane in 3 ml of methanol was added to the above mixture and incubation continued for another 60 min. The solvent was evaporated *in vacuo*, the residue dissolved in chloroform and extracted three times with 0.1 M aqueous sodium bicarbonate. Organic phase was evaporated *in vacuo* and the product purified by silicagel column chromatography using hexane/acetone (3:1) as eluent. Yield—70 mg. ^1^H NMR chemical shifts (d) in DMSO are: 1,2-1.8 (m, 12H,), 1,93 (q, 2H, ζ-methylene, J = 7.2), 3.00 (q, 2H, α-methylene, J = 7.2) 3.72 (q, 2H, 3-methylene, J = 2.4), 4.38 (q, 2H, 4-OC**H**_2_CH_3_, J = 7.2), 5.85 (2H, broad, 7 amine), 6.89 (dd, 1H, 8H, J_1_ = 10, J_2_ = 2.4), 7.17 (t, 3H, p-ArH, J = 7.2), 7.28 (t, 6H, m-ArH, J = 7.4), 7.39 (d, 6H, o-ArH, J = 7.4), 7.67 (m, 1H, 5H), 7.84 (t, 1H, amide, J = 7.2)
    3.9.4 7-amino-4-ethoxy-3-carboxamido(6-isothiocyanobutyl)-2-trifluoromethylquinoline (XX)
      The product **XIX** was dissolved in 2 ml of 90 % acetic acid. After incubation (90 °C, 15 min) the mixture was evaporated *in vacuo*. The residue was suspended in 2 ml of 0.1 M aqueous HCl and extracted with chloroform. The aqueous phase was supplemented with NaOH to pH 12–12.5 and extracted by chloroform. The organic phase was dried over anhydrous sodium sulfate and evaporated *in vacuo*. The residue was dissolved in 2 ml of chloroform/methanol (5:1) mixture and 22 mg (120 μmol) of 1,1′-thiocarbonyldiimidazole were added. After 10 min, 150 μmol of trifluoroacetic acid was added and incubation continued for 60 min at 50 °C. The mixture was diluted with water and extracted with chloroform. The organic layer was evaporated and the product purified by silicagel column chromatography. Yield: 40 mg. ^1^H NMR chemical shifts (d) in DMSO are: 1.2–1.5 (m, 9H), 1.62 (m, 2H, ε-CH_2_, J = 7.2), 3.04 (m, 2H, α-CH_2_, J = 7.2), 3.65 (t, 2H, ζ-CH_2_, J = 7.2), 3.73 (s, 2H, 3-methylene), 3.90 (q, 2H, -OC**H**_2_CH_3_, J = 7.2), 5.85 (s, 2H, broad, 7 amine), 6.7 (d,1H, 8H, J = 2.4), 6.89 (dd, 1H, 6H, J_1_ = 7.2, J_2_ = 2.4), 7.66 (m, 1H, 5H), 7.87 (t, 1H, broad, amide).
  3.10 Click reactions.
    Three microliters of 2 M triethylammonium acetate buffer, pH 7.0 and 15 μl of DMSO were mixed with 5 μl of 0.5 mM alkyne-modified oligonucleotide possessing, or lacking a quencher. One microliter of the 10 mM azido-fluorophore, 3 μl of 5 mM ascorbic acid solution; and 3 μl of 10 mM Copper (II)—TBTA stock in 55 % DMSO, were consequently added to the solution. After overnight incubation at room temperature, the mixture was supplemented with 3 μl of 3 M NaAc pH 5.5 and oligonucleotide material precipitated by addition of 300 μl of ethanol. The residue was additionally washed by 80 % ethanol (2 × 0.3 ml), dissolved in water and subjected to reverse phase HPLC.
  3.11 Reaction of isothiocyano-compound with cysteine and ethylenediamine.
    The reaction mixture contained 0.1 M sodium-borate pH 9.5, 10 mM cysteine, and 0.1–1 mM of isothiocyano compound. The mixture was incubated 5 min at room temperature and the reaction products analyzed by TLC using acetonitrile-water (3:1) as developing solvent. The reaction with ethylenediamine was performed with 0.1 M ethylenediamine pH 8.0 at 50 °C for 1 h. The products were analyzed as described for cysteine reaction.

## Electronic Supplementary Material

Below is the link to the electronic supplementary material.

ESM 1 (DOCX 195 kb)
