# Supplementary material for: New Cross-Linking Quinoline and Quinolone Derivatives for Sensitive Fluorescent Labeling
Source: J Fluoresc. 2012 Mar 28;22(4):1021–32. doi: 10.1007/s10895-012-1039-z (PMC3397220; doi:10.1007/s10895-012-1039-z)
Supplement: Supplementary file 1 — (DOCX 195 kb) [file 10895_2012_1039_MOESM1_ESM.docx]

**Supplementary material.**

New Cross-linking Quinoline and Quinolone Derivatives for Sensitive Fluorescent Labeling.

Shyamala Pillai^†‡^, Maxim Kozlov^‡§^, Salvatore A. E. Marras^‡^, Lev N. Krasnoperov^†^,
and Arkady Mustaev^‡*^.

^†^Department of Chemistry and Environmental Sciences, New Jersey Institute of Technology, 151 Tiernan Hall, University Heights, Newark, New Jersey 07102, USA.

^‡^PHRI Center, New Jersey Medical School, Department of Microbiology and Molecular Genetics, University of Medicine and Dentistry of New Jersey, 225 Warren Street, Newark, New Jersey 07103, USA

^§^Enghelhardt Institute for Molecular Biology RAN Moscow, Vavilova 32, Russia 117984

*To whome correspondence should be addressed

E-mail: [mustaear@umdnj.edu](mailto:mustaear@umdnj.edu)

Tel. 973-854-3442

Fax: 973-854-3101

Figure S1. Time course diagram for product accumulation in the reaction mixture containing 1.3-phenylenediamine and trifluoroacetoacetate. Products are numbered according to Scheme 1.

Figure S2. HPLC analysis of the reaction mixture after click-attachment of fluorophore **X** to a DNA oligo. A = Fraction of DNA oligo without fluorophore conjugated; B = Fraction of DNA oligo with fluoropfore label.
